# Delineation of three-dimensional tumor margins based on normalized absolute difference mapping via volumetric optical coherence tomography

**DOI:** 10.1038/s41598-024-56239-3

**Published:** 2024-04-05

**Authors:** Jae-Sung Park, Taeil Yoon, Soon A. Park, Byeong Ha Lee, Sin-Soo Jeun, Tae Joong Eom

**Affiliations:** 1 Department of Neurosurgery, Seoul St. Mary’s Hospital, College of Medicine, The Catholic University of Korea, 222 Banpo-daero, Seocho-gu, Seoul, 06591 Republic of Korea; 2 School of Electrical Engineering and Computer Science, Gwangju Institute of Science and Technology (GIST), Gwangju, Republic of Korea; 3 Department of Biomedicine and Health Science, College of Medicine, The Catholic University of Korea, Seoul, Republic of Korea; 4 Department of Cogno-Mechatronics Engineering, Pusan National University, Busan, 46241 Republic of Korea; 5 Engineering Research Center for Color-Modulated Extra-Sensory Perception Technology, Pusan National University, Busan, 46241 Republic of Korea

**Keywords:** Glioblastoma, Swept-source optical coherence tomography, Biomedical optics, Optical signal attenuation, Intraoperative diagnostic techniques, CNS cancer, Imaging and sensing

## Abstract

The extent of surgical resection is an important prognostic factor in the treatment of patients with glioblastoma. Optical coherence tomography (OCT) imaging is one of the adjunctive methods available to achieve the maximal surgical resection. In this study, the tumor margins were visualized with the OCT image obtained from a murine glioma model. A commercialized human glioblastoma cell line (U-87) was employed to develop the orthotopic murine glioma model. A swept-source OCT (SS-OCT) system of 1300 nm was used for three-dimensional imaging. Based on the OCT intensity signal, which was obtained via accumulation of each A-scan data, an *en-face* optical attenuation coefficient (OAC) map was drawn. Due to the limited working distance of the focused beam, OAC values decrease with depth, and using the OAC difference in the superficial area was chosen to outline the tumor boundary, presenting a challenge in analyzing the tumor margin along the depth direction. To overcome this and enable three-dimensional tumor margin detection, we converted the *en-face* OAC map into an *en-face* difference map with x- and y-directions and computed the normalized absolute difference (NAD) at each depth to construct a volumetric NAD map, which was compared with the corresponding H&E-stained image. The proposed method successfully revealed the tumor margin along the peripheral boundaries as well as the margin depth. We believe this method can serve as a useful adjunct in glioma surgery, with further studies necessary for real-world practical applications.

## Introduction

Intrinsic human brain gliomas infiltrate to the adjacent brain tissues. T2 or FLAIR (fluid-attenuated inversion recovery) images can reveal tumor cells in a high signal intensity zone; these cells are also detected in the well-enhanced regions of a gadolinium-enhanced T1 image^1^. Glioblastomas, which are the most aggressive form of malignant gliomas, are associated with negative prognosis. Even with the best multidisciplinary treatments, the average expected survival is 14.6 months^2^. Among several prognostic factors that extend survival, the extent of surgical resection has a good prognostic value in patients with initially diagnosed glioma^3–6^, as well as in those with recurrent disease^3,7,8^. The postoperative MRI scan can reveal tumor remnants even in cases of gross total tumor removal intraoperatively^9^. Supra-total or supra-marginal resection has led to promising OS (overall survival) and PFS (progression-free survival) in selected patients^10,11^. However, when the tumor is located in the eloquent area of the brain, the extent of resection is limited to preserve the neurocognitive function.

Various intraoperative imaging techniques are currently utilized to facilitate maximal safe resection. Intraoperative CT, MRI, or ultrasound are used in combination with a navigation system^12–18^. Supplementary methods, such as fluorescence-guided surgery (FGS) using 5-aminolevulinic acid (5-ALA), have shown positive results^13,19,20^, and has been used in selected patients. The United States Food and Drug Administration approved 5-ALA in 2017, and ever since, it is widely used in surgery for glioma patients^21^. The positive predictive value of 5-ALA in FGS for glioma surgery was close to 100%, but the negative-predictive value was not found to be high due to the infiltrative nature of HGGs. The benefit on extent of resection (EOR) was shown in image guided surgery, including the use of intraoperative MRI and 5-ALA, but the evidence quality was very low^13^. Awake surgery with appropriate real-time neurocognitive monitoring has been increasingly advocated for maximal tumor resection with acceptable or no surgical morbidities^22–24^.

Other recent advanced techniques that are used for intraoperative neurosurgical guidance include optical imaging methods, such as diffuse reflectance spectroscopy, optical coherence tomography (OCT), and Raman spectroscopy^25^. Several methods have been developed to distinguish tumor cells from normal tissue by using OCT^26^, enabling non-invasive tomographic imaging. Lenz et al. and Möller et al. introduced a texture-based classification algorithm for ex-vivo OCT images to differentiate healthy tissue from meningioma and adult-type glioma WHO grade 4, respectively^27,28^. However, because this method does not consider the biological and physical characteristics of actual tissues, its applicability in clinical practice remains uncertain. Also, neither study provided research results distinguishing the margin of tumors growing within normal tissue. Fan et al. introduced a study on tumor boundary detection using a deep learning model^29^. However, the mask used as the ground truth is manually drawn, leading to unclear accuracy, and there is a drawback that new training may be required depending on the shape and size of the tumor. Gesperger et al. introduced a hybrid prototype that combines OCT with 5-ALA fluorescence for diagnostic imaging^30^, while Kut et al. revealed that there were differences in the optical attenuation coefficient (OAC) between normal tissue and tumors^31^. Both studies utilized absolute OAC values to distinguish between normal tissue and tumors. Nonetheless, limitations arose due to the severe decrease in the OAC values along the depth direction^32^. This limitation hindered the differentiation in deeper areas. Consequently, only the OAC difference in the superficial area was employed to delineate the tumor boundary, which means analyzing the tumor margin along the depth direction remains challenging^31^.

In this study, we present a groundbreaking analysis focused on the three-dimensional detection of tumor margins. This analysis is based on the OAC difference, as measured by OCT, between normal tissue and tumor cells. The primary objective is to assess the feasibility of glioma removal surgery. To accomplish this, we utilized a swept-source OCT (SS-OCT) imaging system, which was optimized to mitigate OCT signal changes associated with unwanted interferometer movement as reported in a prior study^31^. Our exploration aimed to discern whether volumetric information concerning the tumor margin could be distinguished from the surrounding normal tissue in an orthotopic glioma mouse model. Through the utilization of our proposed normalized absolute difference (NAD) map, we successfully derived the three-dimensional peripheral boundary and depth margin of the tumor.

## Results

### Study design for delineation of three-dimensional tumor margins

**Figure 1:**
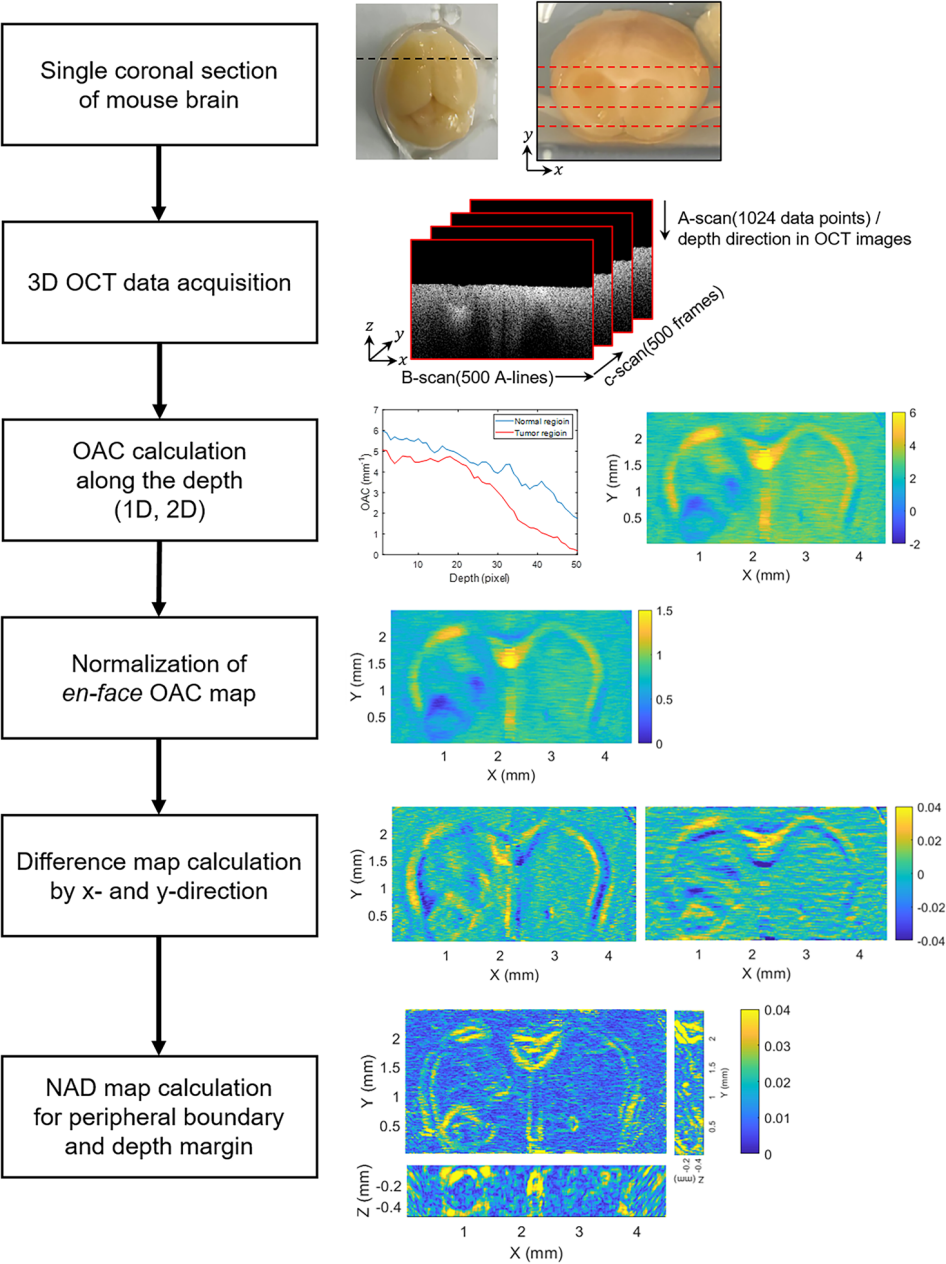
Study design for delineation of three-dimensional tumor margins based on normalized absolute difference mapping via volumetric optical coherence tomography. A single coronal section of mouse brain was performed along the black dashed line and a B-scan images was acquired along the red dashed line. OAC: optical attenuation coefficient, NAD: normalized absolute difference.

To establish a glioma mouse model, orthotopic implantation of human glioma cell line (U-87 MG) was performed. The detailed description regarding sample preparation is presented in the “Methods” section “Sample preparation”. Due to the limited penetration depth of the OCT system^33,34^, imaging the tumor through the outer surface of the mouse brain posed a challenge. Instead, we performed a coronal section only once based on the needle mark where the glioma cell was injected, and then fixed the cross-section to ensure that it was perpendicular to the laser incidence of the OCT system, as shown in Fig. 1. A single coronal section was performed along the black dashed line on the extracted mouse brain, and a total of 500 B-scan images were acquired along the red dashed line, forming a three-dimensional volumetric OCT dataset. To obtain the three-dimensional peripheral boundary and depth margin of the tumor, we initially calculated the OAC from a single coronally sectioned brain surface. Subsequently, we reconstructed an *en-face* OAC map at a specimen depth using the same volumetric OCT dataset. To compensate for the OAC attenuation in the depth direction, normalization was performed, ensuring constant OAC of the tumor region at each depth in the *en-face* OAC maps. By varying the depth in a stepwise fashion, we reconstructed and normalized a series of *en-face* OAC maps. With this series of *en-face* normalized OAC (NOAC) maps, a three-dimensional NAD map was obtained by calculating the root-mean-square (RMS) value of NOAC difference in both x- and y-directions. The detailed calculation processes and results for each step are described in the following sections.

### Calculating the OAC along the depth direction

**Figure 2:**
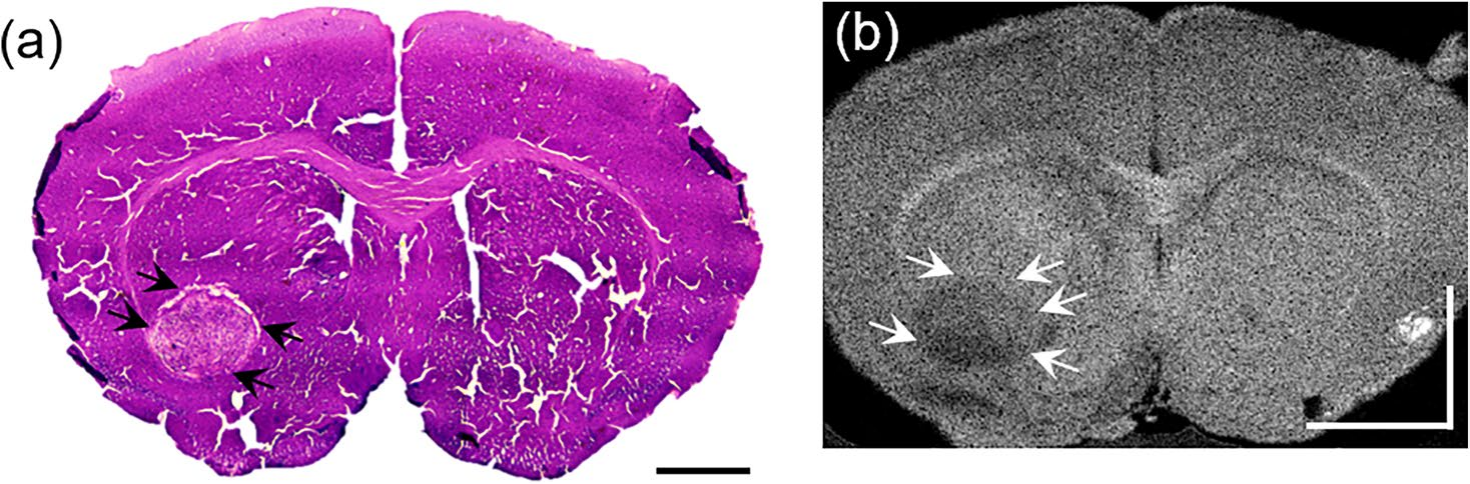
Comparison of H&E-stained and OCT *en-face* images. A tumor boundary was obtained as indicated by the black arrow in the H&E-stained image (**a**) and a white arrow in the OCT *en-face* image (**b**). Black and white scale bar: 1 mm.

**Figure 3:**
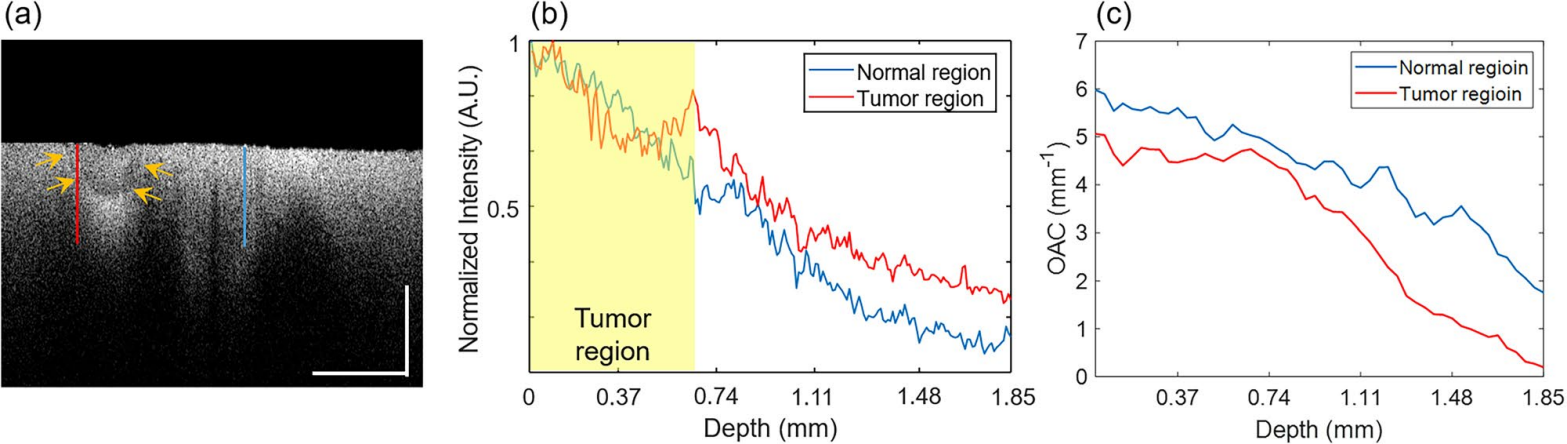
OCT B-scan image (**a**) and its normalized intensity changes in depth of the normal tissue (blue solid line) and tumor (red solid line) region (**b**). (**c**) The OAC depth profiles were calculated by moving the window of 375 µm, corresponding to 40 pixels in the depth direction from the surface. White scale bar: 1 mm.

The 3D volumetric OCT data were acquired from the extracted mouse brain with dimensions of 4.8 mm (1024 data points/A-line) × 4.5 mm (500 A-lines/frame) × 4.5 mm (500 frames/volume) using the 1300 nm wavelength SS-OCT (Supplementary Fig. S1). Figure 2 shows an H&E stain image alongside an acquired *en-face* OCT image of the coronally sectioned mouse brain utilized in the experiment. The tumor region indicated by the black arrow in Fig. 2a appears darker than the normal tissue, as indicated by the white arrow in Fig. 2b. However, in the grayscale *en-face* OCT image in Fig. 2b, although there exists intensity difference between the two regions, the tumor's peripheral boundary remains unclear. To compare the optical scattering characteristics, the OCT signal profiles were analyzed at the surface in the depth direction as shown in Fig. 3.

Figure 3a shows a cross-sectional (B-scan) image in the area where the tumor exists as was indicated by the white arrows in Fig. 2b. The signal intensity in the tumor area is low, similar to the OCT *en-face* image in Fig. 2b. To compare the signals at a same depth, the location of the surface was identified first. In Fig. 3a, we can see that the OCT signals are resulted from both the surface and the interior of the sample. However, the most significant change is observed at the surface. Consequently, by tracking the position of the greatest OCT intensity variation along each A-lines, the surface profile could be identified. The normalized profiles of logarithm of OCT signal in the depth direction, taken along the tumor region (red solid line) and the normal tissue region (blue solid line), are presented in Fig. 3b. Interestingly, up to a depth of 0.65 mm, the signal intensity in the tumor region was attenuated more slowly compared to the normal tissue, as indicated by the yellow box in the Fig. 3b. However, from 0.65 to 1.85 mm, both lines exhibited a similar gradient. This similarity in the slope indicates that beyond the tumor margin, there were normal tissues along both lines. In order to numerically compare the degree of signal attenuation, the slope of the OCT signal intensity was calculated first. We calculated the OAC value spanning 375 μm in the depth direction, corresponding to 40 pixels, through linear fitting of the logarithm of the exponentially decaying OCT signal (“Methods” section “Calculation of NAD”). By moving the window of 40 pixels in the depth direction, the OAC depth profile could be obtained as Fig. 3c. We clearly see that the OAC value decreases with depth but becomes small in the tumor region compared to the normal tissue region. This indicates a limitation of using absolute OAC value to differentiate between normal tissue and tumor.

### Normalization of OAC around tumor region

**Figure 4:**
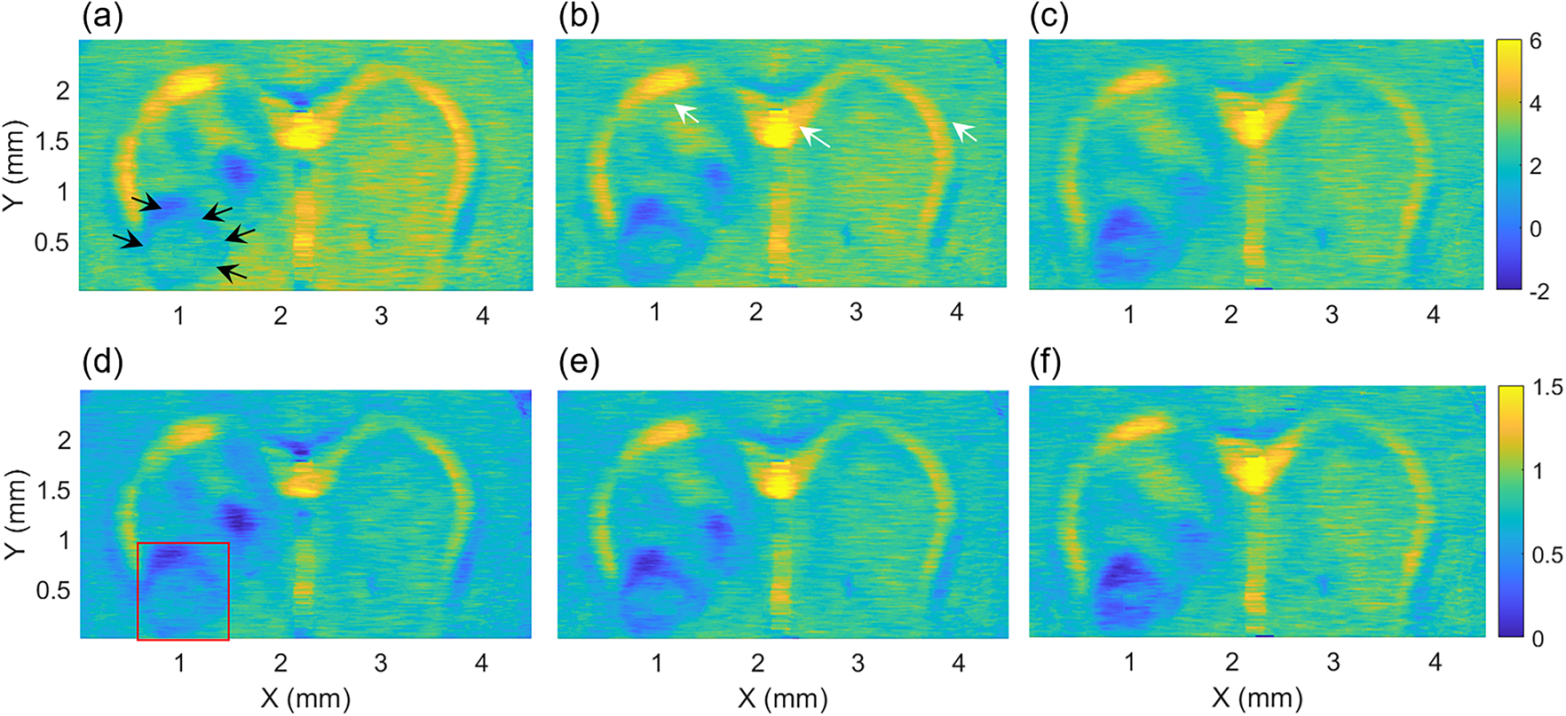
Comparison of original and *en-face* NOAC maps along the depth. The *en-face* OAC map calculated at depths of (**a**) 405, (**b**) 450, and (**c**) 495 μm from the surface of brain, respectively. Colorbar: mm^−1^. Based on OAC in the tumor region indicated with the red box, the *en-face* OAC maps were normalized at depths of (**d**) 405, (**e**) 450, and (**f**) 495 μm, respectively.

To determine the peripheral boundary and the depth margin of the tumor, the OACs were calculated for 500 A-lines that make up one B-scan image, and after calculating OACs for all 500 B-scans, an *en-face* OAC map consisting of 500 × 500 pixels was obtained as shown in Fig. 4. Figure 4a–c represent the OAC maps calculated at depths of 405, 450, and 495 μm, respectively. Each pixel of the *en-face* OAC map indicates the OAC value calculated along each A-line over 40 pixels, depicted with the colorbar. The area indicated by the black arrows in Fig. 4a is the tumor region, where we can see that the OAC was smaller than normal tissue region, and the tumor boundary shrinks as the depth increases. However, in Fig. 4c, we can see that OAC becomes smaller in both the tumor and the normal tissue region than in Fig. 4a. Thus, we can say that due to the attenuation of OCT intensity with depth, the OAC and the contrast of the tumor boundary are decreased with depth. Interestingly, the corpus callosum region, indicated by white arrow in Fig. 4b, showed a high OAC value at all depths.

To enhance the contrast of the tumor boundary, we normalized the *en-face* OAC map for each depth by using the maximum and minimum OAC values within the red box in Fig. 4d, corresponding to the tumor region. This means that despite changes in depth, the OAC values within the tumor region are maintained between 0 and 1. By ensuring the consistency of the *en-face* NOAC maps at different depths, as shown in Fig. 4d–f, a coherent depiction of the tumor boundary was achieved with enhanced contrast (Supplementary Fig. S2). This approach allowed for a more precise and visually impactful representation of the tumor boundary in relation to its depth variation.

### Calculation of normalized absolute difference (NAD) map

The *en-face* NOAC map provides accurate OAC value and indicates the location of the tumor well. This can be a useful indicator for determining the tumor boundary because the contrast between the tumor and normal tissue is higher than that of the conventional gray-scale OCT intensity image.

**Figure 5:**
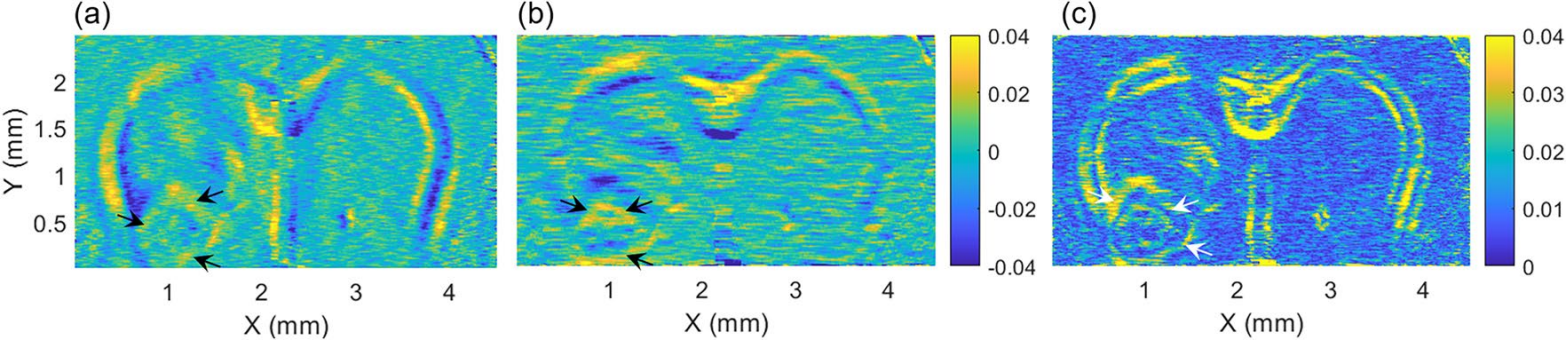
Calculation of OAC differences in (**a**) *x*- and (**b**) *y*-directions. (**c**) NAD map derived from the RMS of (**a,b**). The peripheral boundary of tumor was clearly distinguished from the normal tissue region.

To maximize the difference, for visualizing the peripheral boundary of the tumor, the OAC difference between adjacent two pixels was utilized. To exclude small variations in the *en-face* NOAC map, low-pass filtering was applied in both *x*- and *y*-directions before taking the OAC difference. Considering the round shape of the tumor, as was identified in the OAC map, the differences $\Delta NOA{C}_{{x}_{i}}$ and $\Delta NOA{C}_{{y}_{j}}$ along both directions were calculated and presented in Fig. 5a, b (“Methods” section “Calculation of NAD”). The difference was large at the tumor boundary, as indicated by the black arrows in Fig. 5a, b. Since the differences could be negative and positive, the RMS was calculated. Figure 5c shows the RMSs taken in both directions. Compared with the conventional *en-face* OCT image of Fig. 2b, we can see that the peripheral boundary of the tumor in Fig. 5c shows higher contrast as indicated with white arrows, except for structural differences in corpus callosum. The H&E-stained image presented in Fig. 2a and the NAD map are not completely identical because it is unlikely to obtain the exact same and disfiguration of the sample during staining. However, the resemblance between the images is clear.

### Tumor identification along the peripheral boundary and depth margin

**Figure 6:**
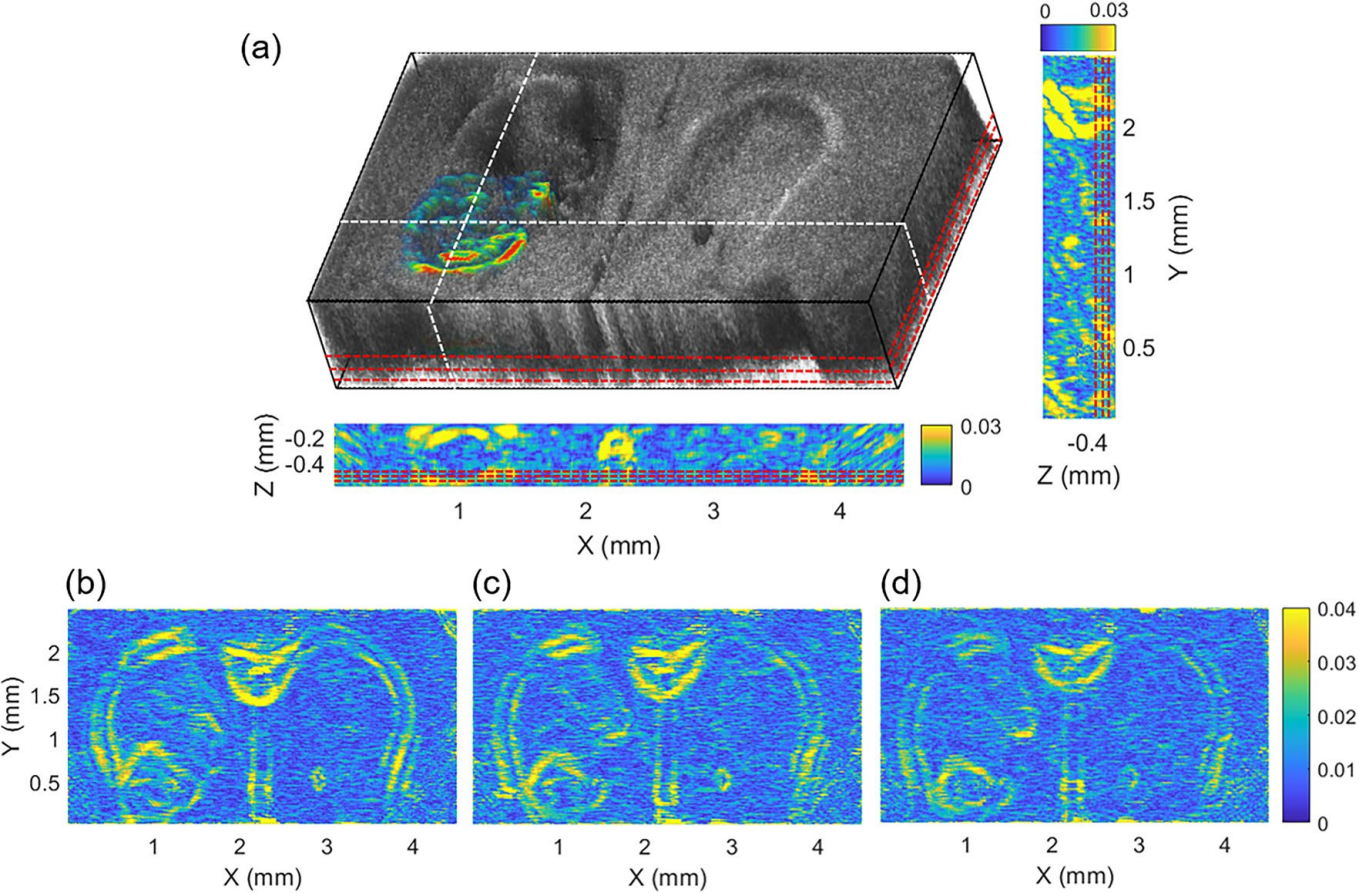
Volumetric NAD map and its cross-sectional and *en-face* images. (**a**) Volumetric NAD map around tumor region and gray-scale OCT image were overlaid and XZ and YZ and cross-sectional NAD map were obtained along the white dotted lines. Also, *en-face* NAD maps at depths of (**b**) 468, (**c**) 522, and (**d**) 558 μm were obtained along the red dotted lines, respectively.

To determine the depth-specific peripheral boundary and the tumor depth margin, we computed the NAD at each depth and constructed a volumetric NAD map, as presented in Fig. 6. Figure 6a shows the surface-segmented gray-scale volumetric OCT image overlaid with the volumetric NAD map around the tumor region. The contrast of the tumor boundary, which was difficult to distinguish due to the low-intensity difference in the gray-scale OCT image, has increased and matches well. Further, the XZ and YZ cross-sectional NAD maps, taken along the white dashed lines, reveal distinct depth margins, compensating for the attenuated OAC values in depth via normalization (Supplementary Fig. S3). Along the red dashed lines corresponding to depths of 468, 522, and 558 μm, the *en-face* NAD maps were taken and shown in Fig. 6b–d, which demonstrate the effective differentiation of the tumor peripheral boundary at all depths. In other words, it is evident that as the depth increases, the tumor boundary shrinks, leading to the determination of the tumor margin consistent with the observations presented in Fig. 6a (Supplementary Video S1).

## Discussion

Brain tumors can be largely categorized into extra-axial and intra-axial tumors. The extra-axial tumors such as meningiomas and schwannomas are relatively well-demarcated and tumor dissection is done readily under a microscopic view. By contrast, intra-axial tumors are embedded into normal brain tissues and the dissection plane can be vague. The most popular intra-axial tumors include metastatic brain tumors and gliomas. The cellular origin of metastatic brain tumors can be traced to outside the CNS, with a relatively clear boundary between the tumor and adjacent normal tissue. Despite tumor invasion, they exhibit a relatively shallow depth^35^. By contrast, in gliomas, the boundary between tumor and the surrounding normal tissue can be more complex. Tumor cell infiltration extends beyond several centimeters of highly cellular tumor margins^1^. Further, the eloquent areas are more directly invaded than other tumorous pathologies. These findings make total tumor removal almost impossible in glioma, literally.

To address this challenge, various intraoperative techniques were developed. Intraoperative CT, MRI, and ultrasound were used since the early twenty-first century, mainly due to their cost-effectiveness and rapid results in surgery^14–18^. Fluorescence-guided surgery using 5-ALA or fluorescein was another technique utilized broadly^19,20,36^. The major limitation of fluorescence-guided surgery is the possibility of obtaining false-positive or false negative intraoperative findings, along with drug-related adverse events^36^.

Optical technologies are another strategy. While not actively utilized in current practice, various optical techniques have been investigated by studies for clinical application^25^. Several studies have already focused on the use of OCT technique in glioma surgery, with potential benefit for achieving maximal safe resection^31,37–39^. Various OCT techniques including time-domain OCT, spectral-domain OCT, full-field OCT, and SS-OCT have been investigated. It was reported that OCT could be used to differentiate cancer from noncancer tissue by calculating the OAC values intraoperatively in real time.^31^ However, due to intra-tumoral heterogeneity of glioblastoma, increased cellularity can be identified in tumor regions, while decreased cellularity can be observed in the necrotic core with the same specimen^40,41^. Furthermore, using the absolute value of OAC were limited to the superficial area due to the variation of OAC value with depth. This also accounts for the inability to analyze the depth margin before. These facts may result in findings inconsistent with those of OCT.

In our study, instead of quantitative analysis, we intended to delineate the tumor-normal interface in a more intuitive fashion. We used the SS-OCT technique, which was the most recently developed OCT technique with substantially higher image processing capability than the conventional OCT. Rather than digitizing the image data^31^, we distinguished cancer versus noncancer status by improving image discrimination for maximizing the surgeon’s control of the extent of tumor resection intraoperatively. We utilized the volumetric NAD map to easily identify the tumor peripheral boundaries and depth margins.

The volumetric NAD map served as a crucial tool in easily identifying tumor peripheral boundaries and depth margins. In Fig. 1, where the laser is incident perpendicularly to the cut section of the mouse brain, the OAC can only be calculated in the direction of the incident laser. However, through the acquisition of volumetric OCT images via 3D scanning, OAC values for the entire tumor region can be obtained. This enables the computation of NAD, facilitating the identification of tumor boundaries expanding outward in all directions within normal tissue. To the best of our knowledge, this method has not been previously reported and holds potential as a valuable adjunct for glioma surgery when appropriately applied in clinical practice.

However, the preliminary nature of our study involving a murine glioma model is a limitation. The in vivo study of human glioma is limited by insufficient data regarding photo-hazard risk of the SS-OCT. Another technical issue that needs to be addressed is the limited working distance. If the working distance can be extended similar to the optimal working distance (20–30 cm) of the operating microscope, further advanced technologies such as augmented reality techniques can be utilized.

Once the safety issues are addressed and the OCT is utilized clinically, patients with glioma or even benign brain tumors can benefit from this technique. For instance, in patients with meningioma or schwannoma located close to critical neurovascular structures, OCT imaging can be used to identify the precise location of the structures, leading to decreased surgical morbidity.

The identification of normal-tumor boundaries in an intuitive manner has yet to be extensively studied. Our preliminary results based on orthotopic glioma mouse model suggest that the NAD maps can facilitate the determination in a three-dimensional volume. Furthermore, our objective was to illustrate the potential utility of the *en-face* OAC and NAD map in elucidating the hitherto imperceptible three-dimensional tumor margin. Particularly, if the cell density within the tumor, growing amidst normal tissues, is adequate for OAC calculation, and the tumor area is sufficiently large relative to the resolution of the OCT imaging system, we posit that calculating the three-dimensional tumor margin through the volumetric NAD map becomes feasible.

## Methods

### Sample preparation

The human glioma cell line (U-87 MG) was purchased from American Type Culture Collection (ATCC, Manassas, VA). Cells were grown in Dulbecco’s Modified Eagle Medium (DMEM) supplemented with antibiotics (100 unit/mL of penicillin and 100 μg/mL of streptomycin) and 10% fetal calf serum (Gibco BRL, Gaithersburg, MD). All cells were incubated at 37℃ in a humidified atmosphere containing 5% CO_2_. Cells were detached from the culture flask using 0.25% trypsin solution (Gibco BRL) and washed three times with phosphate buffered saline (PBS), followed by centrifugation.

All animal experiments were performed in accordance with institutional guidelines and were approved by the Institutional Animal Care and Use Committee in the Catholic University of Korea (approval no. 2017-0211-05). This guideline is consistent and in accordance with the ARRIVE guidelines. Total number of 10 mice was prepared and sacrificed for the experiment. Male athymic nude mice (5–7 weeks of age; Charles River laboratories, Wilmington, MA) were anesthetized by peritoneal injection of ketamine/xylazine solution (200 mg ketamine and 20 mg xylazine in 17 mL of saline) at a concentration of 0.15 mg/10 g of body weight. The cranium was fixed in a stereotactic frame (Stoelting, Kiel, WI). The skin was treated with povidone iodine solution, and a 2 to 3 mm midline incision was made anterior to the interaural line. A sterilized 25-gauge needle was used to make a 1 mm burr hole in the right frontal bone, 2 mm lateral and 1 mm anterior to bregma. Stereotactic implantation of human glioma cells (2 × 10^5^ U-87 MG cells/5μL in 3 μL of PBS) was done via a 10 μL Hamilton syringe (Hamilton Company) with a 30-gauge needle using a microinfusion pump (Harvard Apparatus, Harvard Bioscience, Inc.) over 5 min into the frontal lobe (2 mm depth from the skull surface). The burr hole was covered with bone wax and the skin was approximated.

As the implanted tumors grow, the mice showed abnormal behaviors or weight loss. The elapsed time was approximately 3 to 4 weeks from tumor implantation. After deep anesthesia, PBS was injected into the animal’s heart to remove the blood. Perfusion fixation was carried out with 4% paraformaldehyde, and the tumor bearing brains were explanted. The tumor-bearing brain was sectioned in the coronal direction along the initial needle puncture site to reveal the tumor and used for OCT imaging. Cryosection was performed using the CM1850 Cryostat (Leica Biosystems, Deer Park, IL) after rapid freezing with liquid nitrogen. The slice thickness was 14 to 16 μm and the sectioning plane was parallel to the initial sectioning for OCT imaging. Finally, standard H&E staining was performed.

### SS-OCT system

We designed the home-made SS-OCT with a light source (Axsun laser, Billerica, United States) having a central wavelength of 1310 nm and bandwidth of 110 nm. The wavelength sweeping rate of the laser was 100 kHz. The laser beam of an average optical power of 7 mW was split into a sample arm and a reference arm through a 90/10 optical fiber coupler. The light entering the reference arm was irradiated on to a mirror through a polarization controller and a collimator, and the light reflected from the mirror was incident on one port of a 50/50 optical fiber coupler through an optical circulator. In the sample arm, the light was irradiated to the sample through a collimator and a 2D galvo mirror for area scanning and backscattered to the other port of the coupler. The two lights from the optical fiber coupler were guided to a balanced photodetector and their interference signal was acquired with a digitizer (Supplementary Fig. S1). The 3D volumetric OCT images were acquired with dimensions of 4.8 mm (1024 data points/A-line) × 4.5 mm (500 A-lines/frame) × 4.5 mm (500 frames/volume). The axial resolution was measured better than 25 μm and the lateral resolution 28 μm.

### Calculation of NAD

In general, the intensity of an A-scan OCT signal $I\left(z\right)$ decays exponentially along the depth^42^ as

1 $$I\left(z\right)\propto {e}^{-2{\mu }_{t}z}$$

where, ${\upmu }_{{\text{t}}}$ is the total attenuation coefficient of the sample medium under imaging and the factor 2 represents the round-trip path of the light in the sample. By using Eq. (1), the attenuation coefficient ${\upmu }_{{\text{t}}}$ can be obtained from the OCT signal $I\left(z\right)$ simply as

2 $$OAC\equiv {\mu }_{t}=-\frac{1}{2}\frac{\mathit{ln}\left(I\left(z\right)\right)}{z}$$

Conventional OCT B-scan images are sensitive to speckle noise, resulting in a considerable amount of high frequency components in the signal. Because OAC involves calculating the slope of the OCT signal, such speckle noise introduces appreciable errors. To minimize this influence, a moving window was applied to the B-scan image, averaging 20 nearby A-lines^31,43^. Then, an *en-face* OAC maps was calculated by calculating the slope of the OCT signal over 40 nearby pixels along the depth. However, as shown in Fig. 4a–c, increasing the depth resulted in a decrease in the OAC value, posing a challenge in discerning the depth margin (Supplementary Fig. S3). Accordingly, the region indicated by the red box in Fig. 4d was defined as the region of interest (ROI), and normalization was performed to ensure that the OAC of the ROI were maintained along the depth as follows:

3 $$NOAC\left({x}_{n},{y}_{m}\right)=\frac{OAC\left({x}_{n},{y}_{m}\right)-{min}_{ROI}}{{max}_{ROI}-{min}_{ROI}} ,$$

where *n* and *m* denote the coordinates of the pixel along *x*- and *y*-directions, respectively. To determine the peripheral boundary and the depth margin of the tumor from the NOAC map, the difference between two nearby points of the NOAC map was calculated in both directions as

4 $$\Delta NOA{C}_{{x}_{i}}=NOAC\left({x}_{i+1}, y\right)-NOAC\left({x}_{i},y\right) \left(i=\mathrm{1,2},\dots ,n-1\right)$$

5 $$\Delta NOA{C}_{{y}_{j}}=NOAC\left(x,{y}_{j+1}\right)-NOAC\left(x,{y}_{j}\right) (j=\mathrm{1,2},\dots ,m-1)$$

By calculating the RMS value of $\Delta NOA{C}_{{x}_{i}}$ and $\Delta NOA{C}_{{y}_{j}}$, finally the NAD map was constructed as

6 $$NAD\left({x}_{i},{y}_{j}\right)=\sqrt{{\left(\Delta NOA{C}_{{x}_{i}}\right)}^{2}+{\left(\Delta NOA{C}_{{y}_{j}}\right)}^{2}}$$

### Supplementary Information

Supplementary Figures.

Supplementary Video 1.

## Data Availability

The datasets generated during and/or analyzed during the current study are available from the corresponding author on reasonable request.
