# Supplementary material for: Delineation of three-dimensional tumor margins based on normalized absolute difference mapping via volumetric optical coherence tomography
Source: Sci Rep. 2024 Apr 5;14:7984. doi: 10.1038/s41598-024-56239-3 (PMC10994936; doi:10.1038/s41598-024-56239-3)
Supplement: Supplementary file 1 — Supplementary Figures. [file 41598_2024_56239_MOESM1_ESM.docx]

**Delineation of three-dimensional tumor margins based on normalized absolute difference mapping via volumetric optical coherence tomography**

Jae-Sung Park^1^, Taeil Yoon^2^, Soon A. Park^3^, Byeong Ha Lee^2^, Sin-Soo Jeun^1,3*^, and Tae Joong Eom^4,5*^

^1^Department of Neurosurgery, Seoul St. Mary’s Hospital, College of Medicine, The Catholic University of Korea, Seoul, Republic of Korea

^2^School of Electrical Engineering and Computer Science, Gwangju Institute of Science and Technology (GIST), Gwangju, Republic of Korea

^3^Department of Biomedicine & Health Science, College of Medicine, The Catholic University of Korea, Seoul, Republic of Korea

^4^Department of Cogno-Mechatronics Engineering, Pusan National University, Busan, South Korea

^5^Engineering Research Center for Color-modulated Extra-sensory Perception Technology, Pusan National University, Busan, South Korea

**Supplementary Figures**


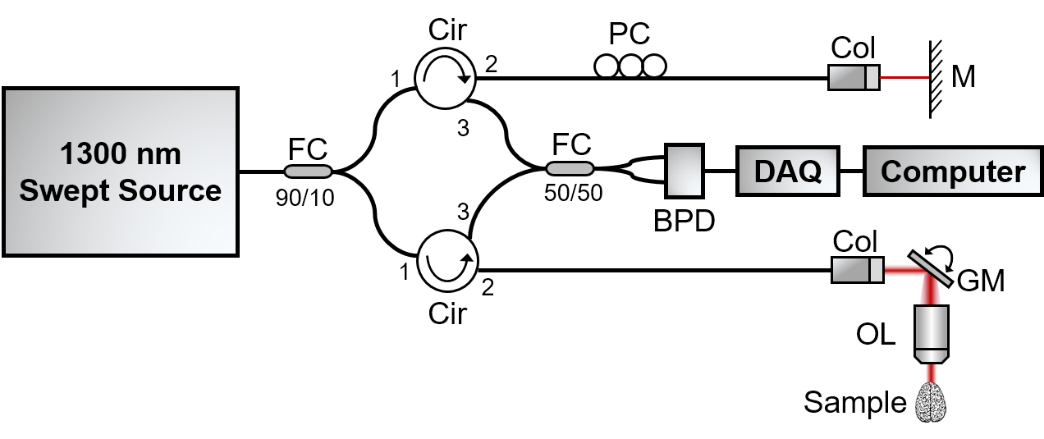


**Supplementary Figure S1 | Schematic representation of experimental setup for SS-OCT.**

FC: fiber collimator, Cir: circulator, PC: polarization controller, BPD: balanced photodetector, GM: galvanometric scanning mirror, DAQ: dispersion compensation plate, OL: objective lens, M: mirror.

**
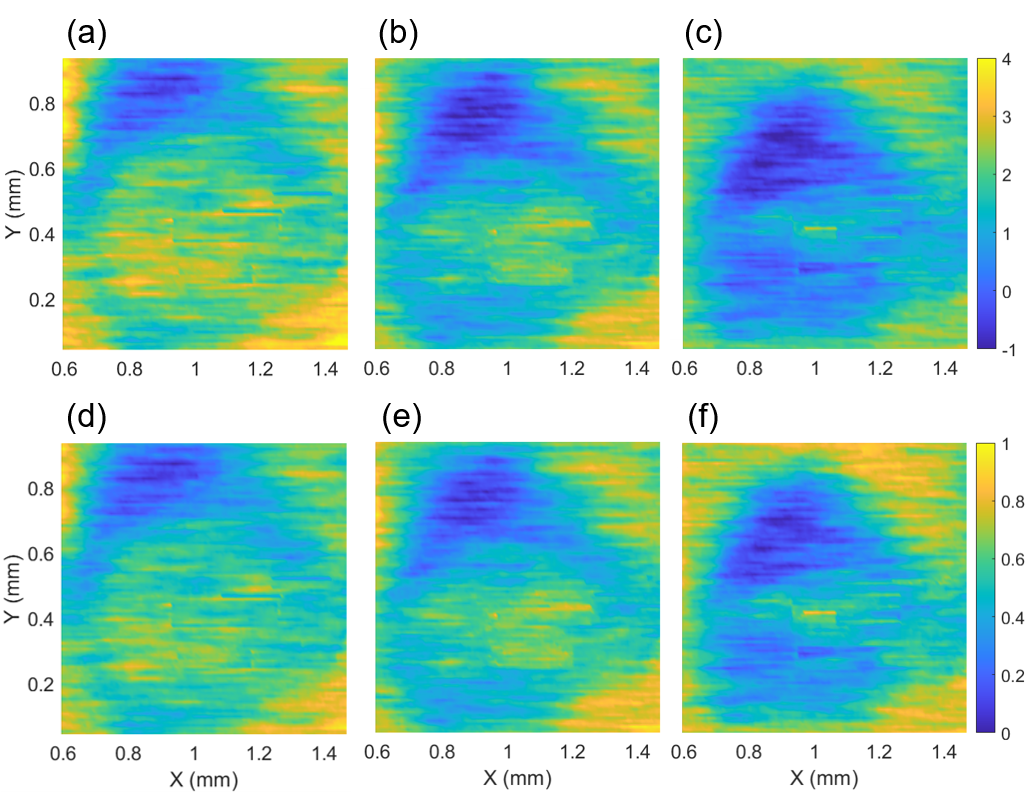
**

**Supplementary Figure S2 | Comparison of enlarged original and *en-face* NOAC maps in tumor region.**

The *en-face* OAC maps and NOAC maps enlarged in tumor region are indicated with a red box in Figure 3(d), at depths of (a, d) 405, (b, e) 450, and (c, f) 495 μm from the surface of brain. (a-c) Absence of normalization decreased the OAC value with increase in depth, while (d-f) normalization ensured a constant OAC value regardless of depth.


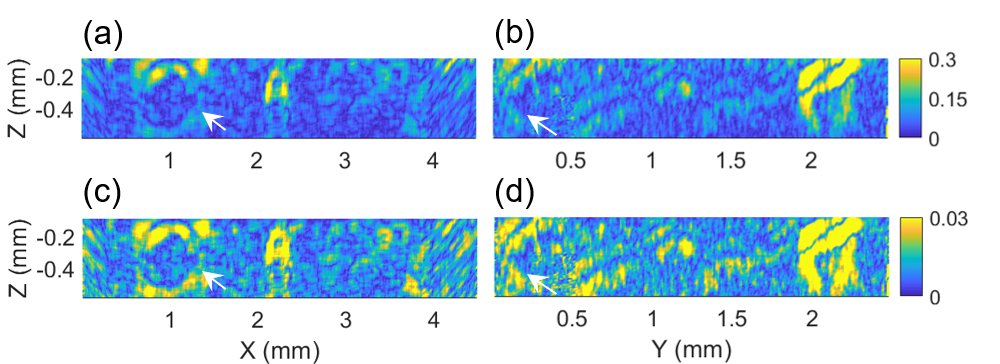


**Supplementary Figure S3 | Comparison of visible tumor depth margin with and without normalization of OAC values.**

(a) and (b) represent cross-sectional absolute difference maps without normalization in the x- and y-directions, and (c) and (d) are cross-sectional NAD maps in x- and y-directions. The tumor margin in the deep region indicated by the white arrow was more clearly identified via normalization.


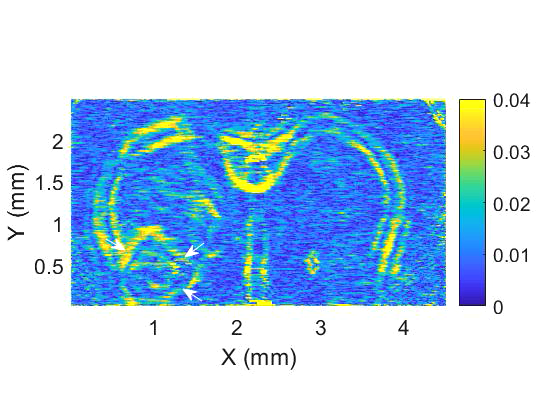


**Supplementary Video 1 | *En-face* NAD maps along the depth direction.**

The tumor margin clearly identified in the deep region also and indicated by the white arrows.
